# Locomotion and attachment mechanisms of the respiratory mite *Orthohalarachne attenuata*

**DOI:** 10.1007/s10493-025-01094-8

**Published:** 2025-12-02

**Authors:** Anika Preuss, David Ebmer, Elena V. Gorb, Adriane Prahl, Michael Flügger, Carlos Hermosilla, Stanislav N. Gorb

**Affiliations:** 1 Department of Functional Morphology and Biomechanics, Zoological Institute, Kiel University, Am Botanischen Garten 1–9, 24118 Kiel, Germany; 2 Vienna Zoo, Maxingstraße 13b, Vienna, 1130 Austria; 3 Institute of Parasitology, Biomedical Research Center Seltersberg (BFS), Justus Liebig University Giessen, Schubertstr. 81, 35392 Giessen, Germany; 4 Tierpark Hagenbeck Gem. GmbH, Lokstedter Grenzstraße 2, 22527 Hamburg, Germany

**Keywords:** Marine mammals, Parasites, Biomechanics, Attachment, Adhesion, Adaptation, Claws, Adhesive pads, Arolium

## Abstract

The respiratory mite *Orthohalarachne attenuata*, a parasite inhabiting the respiratory tract of otariid and odobenid pinnipeds, faces unique biomechanical challenges in its marine environment. It must securely attach to the slippery mucosa of its host while maintaining the ability to traverse dry and wet surfaces to recruit new hosts. This study explores the morphology of the attachment structures and mechanisms of *O. attenuata* larvae using scanning electron microscopy (SEM), confocal laser scanning microscopy (CLSM), high-speed video analysis, and centrifugal force measurements. Our findings confirm that, as in most Mesostigmata, the larvae of *O. attenuata* possess a specialized pretarsal structure consisting of two highly sclerotized claws and a soft, resilin-rich arolium; however, our analyses reveal specific morphological and functional adaptations of this structure related to attachment in the walrus nasal mucosa. Attachment strategy varies with substrate: the claws hook into soft walrus mucosa, whereas the arolium inflates to adhere to smooth surfaces. This dynamic interaction suggests a complex mechanism involving muscle control, flexible cuticle structures, leg positioning, and hemolymph pressure. Larvae employ a tripod gait pattern, facilitating agile movement across diverse surfaces. Force measurements on various substrates (dry and wet hydrophilic glass, hydrophobic plastics, polishing paper of various roughness properties, and walrus mucosa) demonstrate the highest attachment forces on walrus mucosa (safety factor: 348.95 ± 38.70) and wet glass (safety factor: 167.80 ± 119.09), underscoring the critical role of both claws and arolium in securing adhesion. These findings highlight the strong evolutionary adaptations of *O. attenuata* to its challenging environment and offer valuable insights for the development of bioinspired attachment systems suited for soft and wet substrates, particularly in marine applications.

**Supplementary Information:**

The online version contains supplementary material available at 10.1007/s10493-025-01094-8.

## Introduction

Arthropods are the most successful animal phylum, comprising over 80% of all described animal species. Their evolutionary success is reflected in their remarkable diversity in morphology, coloration, and ecological adaptations, allowing them to thrive in a wide range of habitats (Umbers et al. 2014; Aria 2022; Zabeati 2023). The Chelicerata represent a significant subdivision of the Arthropoda, encompassing arachnids and their closest relatives. To date, over 116,000 extant species have been identified, with spiders (Araneae) and mites (Acariformes and Parasitiformes) comprising the majority of the documented taxa (Dunlop 2019). Despite their widespread distribution, relatively few Chelicerata can be found in marine environments (Roth and Brown 1976; Shultz 2001; Leggett et al. 2024). Among these exceptions are members of the family Halarachnidae (Arachnida, Mesostigmata), a small group of mites uniquely adapted to life in aquatic habitats (Furman and Dailey 1980; Alonso-Farré et al. 2012). This family contains, among others, two genera, *Halarachne* (Allman, 1847) and *Orthohalarachne* (Newell, 1947), and five extant species, which infest the respiratory tract of marine mammals as obligate endoparasites (Furman and Dailey 1980; Pesapane et al. 2018, 2021; Shields et al. 2024). While most mites (Acari) are free-living and only some of them are ectoparasites of terrestrial animals (arthropods and vertebrates), these nasal mites reside on the mucosal surface of their host’s respiratory system, feeding on its secretions (gel-like mucus) (Thornton et al. 2008; Alonso-Farré et al. 2012; Reckendorf et al. 2019). However, the two genera have specialized on different hosts: the genus *Halarachne* comprises mites that exclusively parasitize phocids (earless seals) and sea otters, whereas mites of the genus *Orthohalarachne* are parasites of odobenids (walruses) and otariids (sea lions, fur seals) (Domrow 1962; Kenyon et al. 1965).

Their life cycle consists of four stages: a hexapod larva, two short-lived octopod nymphal stages (i.e. proto- and deutonymph), and the adult stage. Notably, the hexapod larval stages are the most mobile ones compared to the other stages, enabling dispersal within and transmission between hosts, whereas nymphs and adults remain mostly sedentary, anchoring themself to the nasal mucosa with specialized tarsal claws (Furman and Smith 1973; Geraci and St. Aubin 1987).

The evolutionary history of Halarachnidae mites is closely intertwined with that of their marine mammal hosts. These parasites likely coevolved with the ancestors of modern pinnipeds during the Oligocene (29–23 million years ago), a period when pinnipeds transitioned from terrestrial to marine environments (Berta et al. 2015; Reckendorf et al. 2019). This shift exposed the parasites to significant ecological challenges, including high hydrostatic pressure, hypoxia, extreme temperature fluctuations, and osmotic stress due to salinity. Over time, Halarachnidae developed specialized behavioral and physiological adaptations to survive in this unique niche (Leggett et al. 2024).

A representative of the genus *Orthohalarachne* is the nasal mite *Orthohalarachne attenuata* Bank, 1910 (Acari; Halarachnidae), which primarily parasitizes California sea lions (*Zalophus californianus*), South American sea lions (*Otaria flavescens*), fur seals (*Arctocephalus spp*.), and walruses (*Odobenus rosmarus*) (Fravel and Procter 2016; Pesapane et al. 2021; Rivera-Luna et al. 2023). Walruses are marine predators that spend almost their entire lives in the sea (Fay and Ray 1968). However, they regularly come ashore during haul-outs to rest and to raise offspring (Fay 1982). Their dives can reach depths of over 100 m and a duration of up to 10.5 min (Fay and Burns 1988; Wiig et al. 1993). During such deep dives, the mite is subjected to an enormous rate of pressure change during ascent and descent and, depending on the depth, a high pressure, which means a pressure of 10 kg*cm^− 2^ (≈1000 kPa) at a depth of 100 m (Leonardi et al. 2020). Nevertheless, *O. attenuata* remains protected inside the host’s nasal passages and is not actively moving during these dives. Continuous attachment to the host tissue is therefore essential to avoid being dislodged by pressure changes or respiratory movements (Fain 1994).

When the host surfaces and comes ashore during haul-outs, however, the ecological context changes substantially. At this stage, attachment and locomotion become critical, as larvae may move within the nasal cavity or between individuals to facilitate transmission and colonization of new hosts (Furman and Smith 1973; Geraci and St. Aubin 1987). The ability to both attach securely to the soft, mucus-covered epithelium and to move efficiently on this viscous surface is thus a key adaptation to their obligate parasitic lifestyle (Fain 1994). The morphology of mite attachment organs reflects this dual functional demand: their structure and diversity are strongly influenced by habitat and lifestyle, with claws typically serving to grasp onto the substrate (Pfingstl et al. 2020; Pfingstl 2023). Studies on representatives of ectoparasitic mites have also shown that they usually have two claws and pad-like structures, presumably to enable adhesion to smooth surfaces (Helle and Sabelis 1985; Evans 1992; Pfingstl 2023). In other arthropods, such pads have been shown to increase friction and enable adhesion to smooth, rough, soft, and hard surfaces (Beutel and Gorb 2001; Gorb and Beutel 2001). However, studies on the function and mode of operation of the mite attachment organs, which could be particularly adapted to the habitat in the soft nasal mucosa, are lacking (Pfingstl 2023).

The aim of this study is to (i) describe the morphology of attachment systems in *O. attenuata* using scanning electron microscopy (SEM) and confocal laser scanning microscopy (CLSM), (ii) analyze the walking patterns of larvae on smooth surfaces using inverse high-speed video recordings and real-time video recordings, and (iii) determine the attachment force on different surfaces, including the physiological nasal mucosa of a Pacific walrus (*O. rosmarus divergens)*, using centrifugal force measurements. In doing so, we shed light on the question of how reliable, strong, and reversible attachment is possible on different surfaces in the marine environment. Furthermore, we provide suggestions for the technical development of biologically inspired attachment systems that also enable locomotion on soft viscous substances.

## Materials and methods

### Mites

Larvae of the respiratory mites (*O. attenuata*; Halarachnidae; Arachnida) (Banks, 1910) were collected during a necropsy of a 28-year-old zoo-held Pacific walrus (*O. rosmarus divergens*) in September 2024. Mites were stored in a nutritive solution (NaCl including mucosa pieces) at ambient temperature (20–22 °C) in a plastic beaker. After force measurements, which took place 5–8 days after the dissection of the host, the mites were stored in 70% ethanol. Ethical review and approval are not required for this study because the host animal died naturally and was not killed for the purpose of this study. We have adhered to all applicable ethical guidelines regarding the use of animals.

### Scanning electron microscopy (SEM)

Larvae of *O. attenuata* (*n =* 5) underwent dehydration using a series of increasing ethanol concentrations, followed by critical point drying in an automated Leica EM CPD300 (Leica, Wetzlar, Germany). The samples were then sputter coated with a 10 nm gold-palladium layer using a Leica Bal-TEC SCD500 (Leica, Wetzlar, Germany). SEM analysis was performed on ventral and dorsal sides of the specimens using a Hitachi TM3000 (Hitachi Ltd., Tokyo, Japan) at 15 kV acceleration voltage. To examine tarsal morphology and visualize contact behavior on smooth surfaces in detail, additional samples (*n* = 2) were analyzed in fresh state using cryo-SEM. This process involved freezing the mites at −140 °C in a cryo stage preparation chamber (Gatan ALTO 2500 cryo preparation system, Gatan Inc., Abingdon, UK). The frozen samples were then observed using a cryo-SEM Hitachi S-4800 (Hitachi High-Technologies Corporation, Tokyo, Japan) at 3 kV accelerating voltage and − 120 °C temperature. The resulting images were processed using Adobe Photoshop CS6 (Adobe Photoshop CS, San José, USA) and Affinity Photo (Serif Ltd, Nottingham, UK).

### Confocal laser scanning microscopy (CLSM)

To prepare mites for CLSM analysis, specimens were immersed in glycerine (≥ 99.5%) and covered with a precision cover slip (thickness = 0.170 ± 0.005 mm, refractive index = 1.52550 ± 0.00015, Carl Zeiss Microscopy GmbH, Jena, Germany) before scanning. A CLSM Zeiss LSM 700 mounted on an upright Zeiss Axio Imager microscope (Carl Zeiss Microscopy GmbH, Jena, Germany) was used to examine the samples’ autofluorescence. The setup utilized four solid-state lasers (wavelengths 405 nm, 488 nm, 555 nm, 639 nm) with corresponding emission filters (BP420–480, LP490, LP560, LP640 nm). Following the method described by Michels and Gorb (2012), less sclerotized cuticle, potentially rich in resilin, was visualized using a 405 nm excitation and a 420–480 nm emission filter. Regions of higher sclerotization were identified using 488 nm and 555 nm laser excitations, with filters allowing emission light above 490 nm and 560 nm, respectively. Extended autofluorescence was captured using 639 nm laser excitation with a 640 nm long-path emission filter. ZEN 2008 software (www.zeiss.de/mikroskopie) and Adobe Photoshop CS6 (Adobe Photoshop CS, San José, USA) were employed to process high intensity projections for qualitative (not quantitative) analysis of cuticle composition (Andersen 1979; Vincent 2002; Michels and Gorb 2012; Büsse and Gorb 2018; Josten et al. 2022). In the autofluorescence images, specific colors correlate with particular material properties (Michels and Gorb 2012) as follows: reddish autofluorescence indicates highly sclerotized cuticle, with more intense red hues suggesting greater sclerotization levels; greenish autofluorescence signifies relatively resilient cuticle with high chitin content; bluish autofluorescence denotes softer, less-sclerotized cuticle areas, often containing resilin.

### Reflection interference contrast microscopy (RICM)

An inverted light microscope (Axio Observer.A1, Carl Zeiss Microscopy, Göttingen, Germany) was employed to observe mite adhesive pads in contact with a glass surface. The internal reflection contrast mode was utilized, wherein the light source is positioned to reflect at the interface of direct contact between the glass slide and the object. This technique results in areas of direct contact appearing as dark spots against a bright background. A cleaned glass coverslip was placed on the stage and examined at ×10 magnification. Mites were allowed to move freely within a small chamber without restraint. The stage was manually adjusted both vertically and horizontally and a high-speed video camera (Photron Fastcam SA1.1, VKT Video Kommunikation, Pfullingen, Germany) captured the contact between pads and glass at 5400 frames per second (fps). Screenshots from the highspeed video were processed using Adobe Photoshop CS6 (Adobe Photoshop CS, San José, USA). An exemplary highspeed video can be found in the Supplementary Material S1.

### Bright-field microscopy

An Olympus SZX10 stereomicroscope (Olympus, Tokyo, Japan) including a SDFPLAPo2XPFC 14-230x magnification objective in brightfield mode was used to take videos of mites (*n* = 5; 25 fps) moving freely in a Petri dish (Rotilabo, 100 × 20 mm; Carl Roth, Karlsruhe, Germany). We used individual snapshots from video sequences for analyzing the movement pattern of mites and processed them in Adobe Photoshop CS6 (Adobe Photoshop CS, San José, USA) and Adobe Illustrator CS6 (Adobe Illustrator CS, San José, USA). An exemplary video can be found in the Supplementary Material S2.

### Mite force measurements

**Fig. 1:**
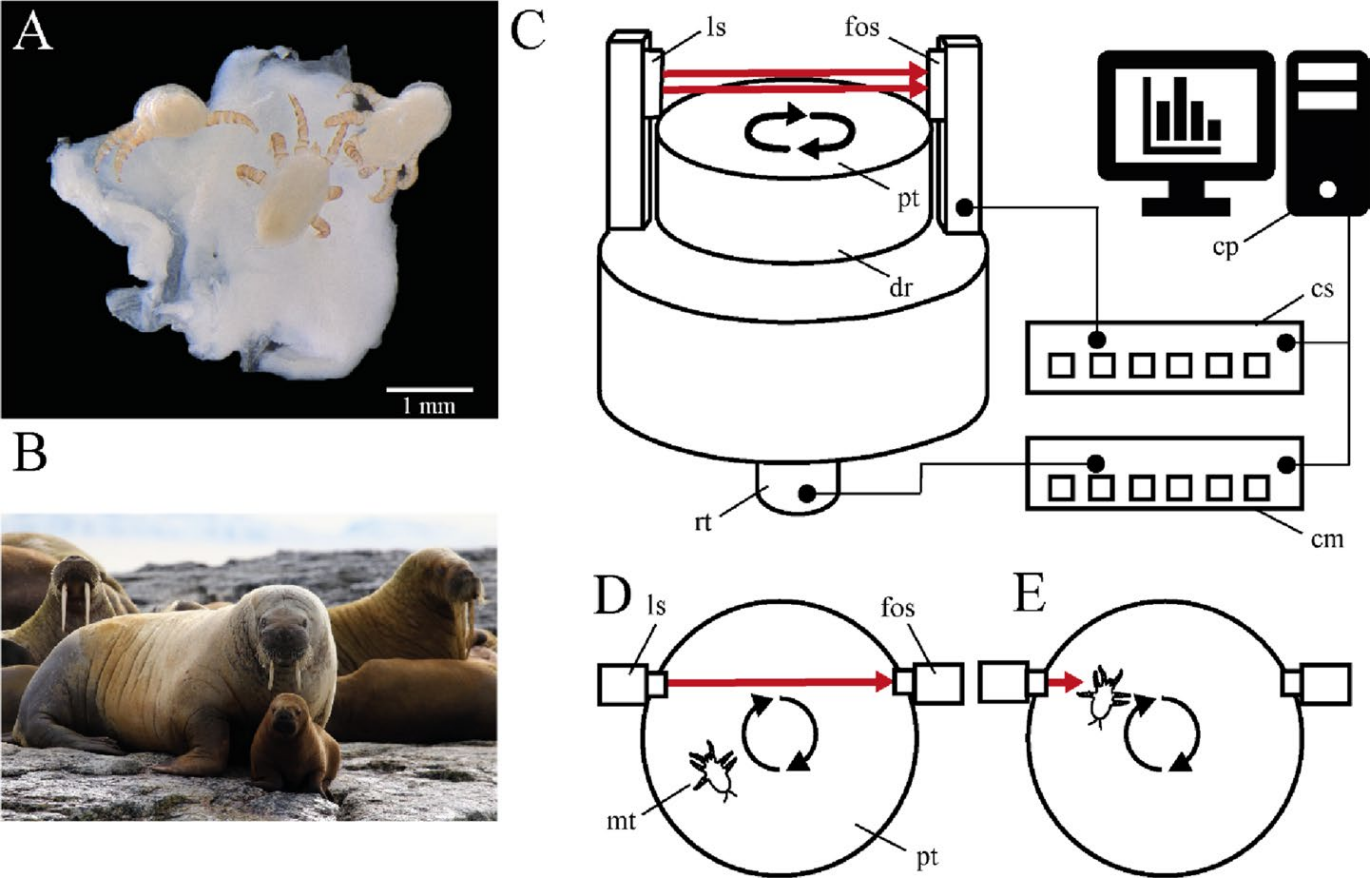
Focal species and experimental setup. (**A**) Hexapod larvae of *O. attenuata* attached to walrus mucosa. (**B**) The host, *Odobenus rosmarus divergens*, *©* West 2023. (**C–E**) Experimental setup for attachment force measurements modified after Gorb et al. 2001 and Gorb and Gorb 2004. The mite (mt) was positioned on a computer controlled centrifugal device equipped with a fibre-optic sensor. (**C**) Scheme of the device. (**D**, **E**) Viewed from above, the scheme illustrates the arrangement of a focused light source (ls, transmitter) and fibre-optic sensor (fos, receiver) in relation to the drum’s center, where a mite rotates on the horizontal drum surface. Additional components include a motor control (cm), computer (cp.), sensor control (cs), drum (dr), Plexiglas plate (pt), and the motor’s rotor (rt)

A centrifugal force tester (Gorb et al. 2001; Gorb and Gorb 2004) was utilized to conduct force measurements on living mites (Fig. 1). A live mite was positioned on horizontal surface of the drum, which rotated at 50·rev·min^–1^ (0.833·rev·s^–1^). The surfaces examined included: (i) 1 μm (roughness) polishing paper, and (ii) 12 μm (roughness) polishing paper, (iii) hydrophilic glass (water contact angle of 67.19 ± 6.14° (mean ± SD), (iv) hydrophobic plastic (transparency film, 110 μm, for laser printers; LMG Germany, Hollenstedt, Germany) (water contact angle of 84.90 ± 6.27° (mean ± SD), (v) hydrophilic glass with water on its surface, and (vi) polished glass with a piece of walrus mucosa whose base had dried on the glass surface to prevent it from slippage. Contact angle measurement data and photos can be found in Supplementary Material S3-S6. The mite’s location on the drum was tracked using a combination of focused light beam and fibre-optic sensor. The drum’s rotational speed was gradually increased from the initial one (50·rev·min^–1^) until the mite lost its grip due to centrifugal force. The maximum frictional component of the attachment force was calculated using the rotational speed at contact loss, the mite’s position on the drum (rotation radius), and the mite’s mass (determined using a Sartorius ultra-microbalance MSE 2.7 S-DM (Sartorius, Göttingen, Germany). Each individual mite (*n* = 15) underwent three repetitions of the experiment on each surface. Force measurement data can be found in the Supplementary Material S7.

### Data analysis and statistics

We analyzed the attachment force of all mites on the different surfaces using a Kruskal-Wallis one-way ANOVA on ranks and a post hoc Dunn’s test, both at a significance level of 0.05, as the data was not normally distributed (criterion for normal distribution: Shapiro Wilk test, *p* > 0.05). All statistical analyses were performed in R studio (R version 4.2.1, the R Core Team 2022). R scripts can be found in the Supplementary Material S8.

## Results

### Morphology of the mite attachment system

**Fig. 2:**
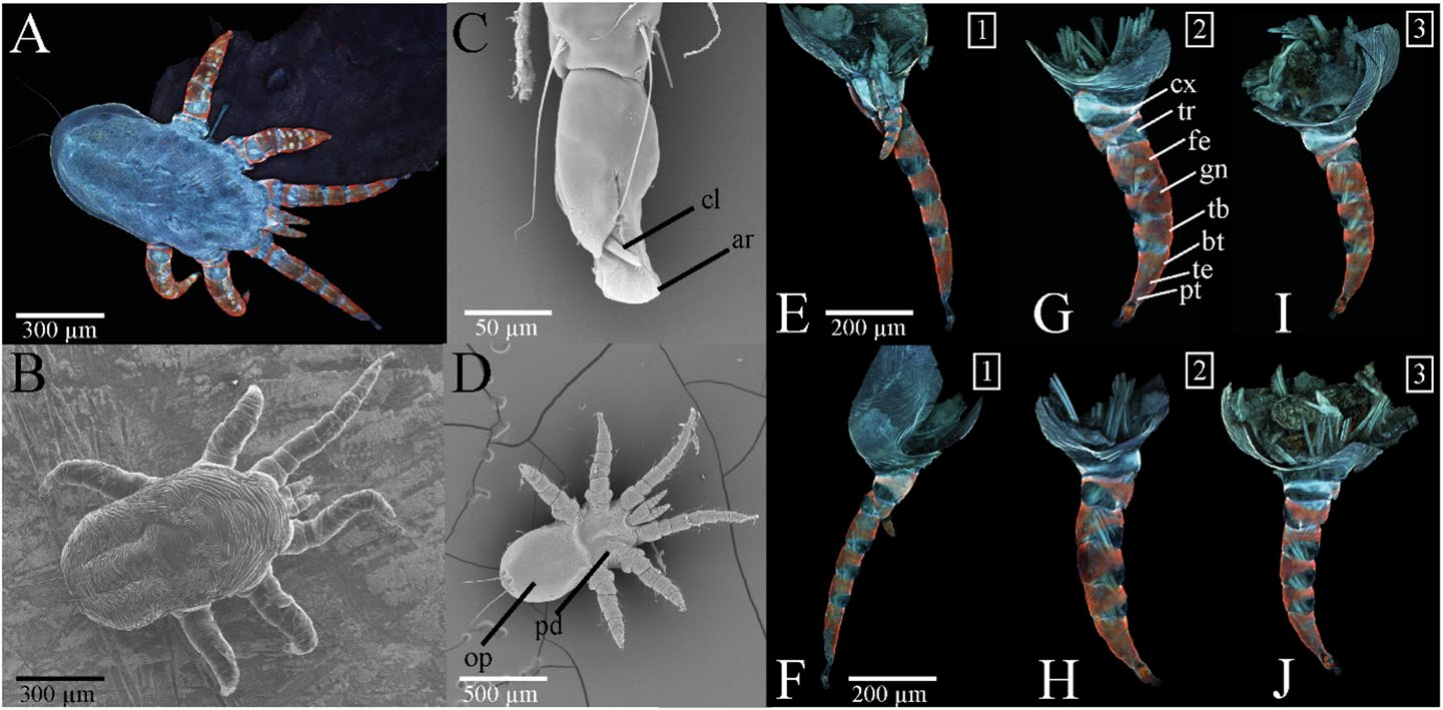
Morphology of the larval leg attachment structures of *O. attenuata*. (**A**) Confocal laser scanning microscopy (CLSM) maximum intensity projection of the entire specimen anchored in walrus mucosa from dorsal view. (**B**-**D**) Scanning electron microscopy image (SEM) of (**B**) the entire specimen on a smooth metal plate frozen at −120 °C in living condition, (**C**) the pretarsus including claw (cl) and arolium (ar) from the middle leg (lateral view), and (**D**) the entire specimen from ventral view. (**E–J**) Confocal laser scanning microscopy (CLSM) maximum intensity projections of (**E**) the first leg (proximal view), (**F**) the first leg (distal view), (**G**) the second leg (proximal view), (**H**) the second leg (distal view), (**I**) The third leg (proximal view), and (**J**) the third leg (distal view). Abbreviations: ar (arolium); bt (basitarsus); cl (claw); cx (coxa); fe (femur); gn (genu); op (opisthosoma); pd (podosoma); pt (pretarsus); tb (tibia); te (telotarsus); tr (trochanter)

The larvae of *O. attenuata* have three pairs of legs originating from the prosoma (Fig. 2A, B & D). A leg consists of eight segments: coxa (cx), trochanter (tr), femur (fe), genu (gn), tibia (tb), basitarsus (bt), telotarsus (te) and pretarsus (pt), whereby the basitarsus and telotarsus together form a single, secondarily divided segment (Fig. 2E–J). The pretarsus is equipped with two claws (cl) and a pad which lies dorsally between the claws (Fig. 2C). We define this pad as an “arolium” following Gorb and Beutel (2001), since the pulvillus in insects is a paired, lateral adhesive organ, whereas the structure observed here is a single, median pad. Similar structures were described by Porta et al. (2024), who examined the ambulacrum of both larvae and adults of *O. attenuata* and reported a pretarsus bearing two claws, a large retractable pad, and fin-shaped paradactyli. While they referred to this pad as a “pulvillus,” we interpret it as a median arolium. The morphological features described by Porta et al. (2024) are consistent with our observations.

When considering the material composition of the cuticle it becomes obvious that all leg segments appear to be highly sclerotized on the dorsal side, while they are less sclerotized on the inner ventral side (Fig. 2A, E–J). Podosoma (pd) and opistosoma (op), on the other hand, are dominated by strong blue autofluorescence and are therefore presumably less sclerotized and thus soft and flexible (Fig. 2A). The pad between the two sclerotized appearing claws is also dominated by blue autofluorescence and probably contains resilin-like proteins responsible for rather soft properties of such cuticle parts (Fig. 2E–J).

Furthermore, it is noticeable that the legs of the mite appear more curved, when it is on a smooth surface, whereas they are more elongated, when it is anchored in the mucosa (Fig. 2A, B).

### Analysis of the attachment posture of *O. attenuata* on rough and smooth surfaces

**Fig. 3:**
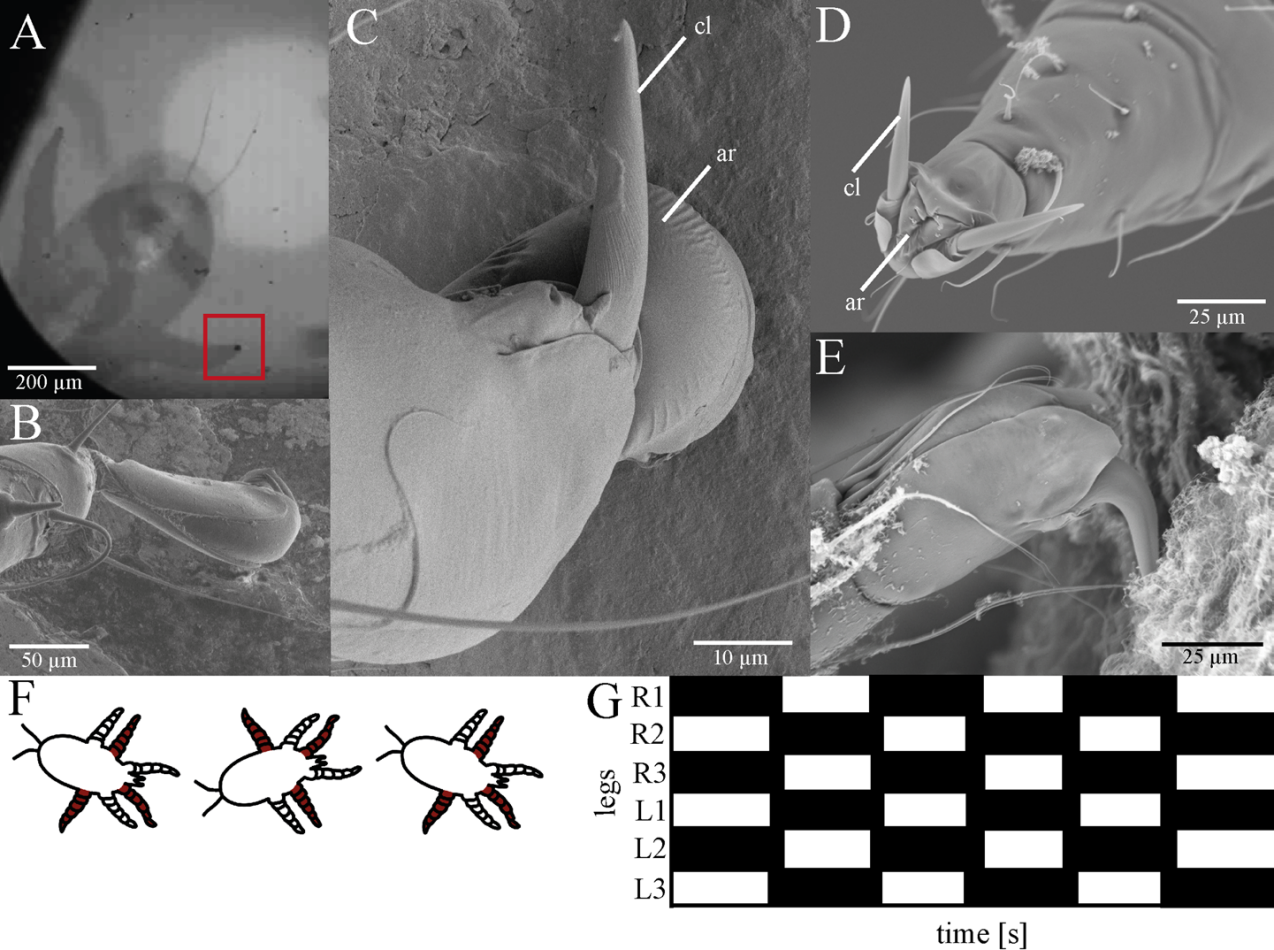
Attachment of *O. attenuata* hexapod larvae on different surfaces. (**A**) Single frame from a high-speed video taken through an inverted reflection contrast interference microscope (RCIM) with an attached adhesive pad highlighted by a red frame. (**B**–**E**) Scanning electron microscopy images (SEM) of (**B**) the first leg placed on a smooth metal surface from dorsal view, (**C**) the second leg placed on a smooth metal surface from dorsal view, (**D**) the pretarsus including claws and inverted arolium from frontal view, and (**E**) the pretarsus with claws hooked into the soft walrus mucosa and inverted arolium. (**F**) Schematic of the walking pattern of a mite with red-labeled legs in contact formation. (**G**) Gait pattern of the larva with black squares indicating that the feet are in a stance phase. Abbreviations: ar (arolium); cl (claw)

When attaching to smooth surfaces, such as glass (Fig. 3A) or polished metal (Fig. 3B and C), the legs of the mite are bent so strongly that its claws are aligned parallel to the substrate surface, but do not touch it. Instead, the arolium between the claws is pressed onto the smooth surface like a stamp, causing it to widen noticeably in contact so that its contact area with the substrate surface is enlarged. This occurs both in the living state, when observed inverted through a glass surface, and in the freshly frozen conditions in the cryo-SEM.

However, when the mite seeks contact with a rough or soft corrugated surface, such as the mucosa of the Pacific walrus, the leg is significantly more elongated and the pretarsus is aligned in such a way that the claws can cling to the soft surface like small climbing hooks (Fig. 3E). On closer inspection, it can also be seen that the arolium appears visually retracted in this type of contact. However, upon careful examination of the pad, it becomes evident that this does not appear to be a method-based drying artifact, as the invagination of the pad appears very regular and does not have the typical irregular surface of a collapsed structure or drying artefact (Fig. 3D).

During our video recordings, both in high-speed mode and at the regular frame rate, we also noticed that the larva uses the tripod-walking pattern typical for insects, in which three legs are anchored to the surface (the middle leg on one side of the body and the front and rear legs on the other side) (Fig. 3F and G). They were able to move nimbly on a wide variety of surfaces, both rough and smooth.

### Attachment force of larvae of *O. attenuata* on different surfaces

Force measurements with larval respiratory mites were performed on six different surfaces: (i) 1 μm roughness, (ii) 12 μm roughness, (iii) hydrophilic glass, (iv) hydrophilic glass with water on its surface, (v) polished glass with a piece of walrus mucosa, and (vi) hydrophobic plastics. After proper contact formation (mite started moving on the surface), the mite was accelerated on the centrifuge until it lost contact to the surface. The maximum measured attachment force averaged over three measurements was 0.680 ± 0.076 mN on mucosa, with the corresponding safety factor 348.95 ± 38.70 (mean ± SD). On wet glass, the maximum attachment force averaged over three measurements was 0.253 ± 0.171 mN with a safety factor of 167.80 ± 119.09. Thereby, the safety factor (calculated as attachment force per body weight) represents the value demonstrating how many own body weights the mite could hold attaching to the different surfaces.

The highest force values (0.39 ± 0.19 mN) were measured on the surface consisting of mucosa attached to glass, with 252.51 ± 105.28 safety factor. Between the mucosa and all other tested surfaces, statistically significant differences were determined in both force value and safety factor (*p* < 0.001). On the glass surface wetted with water, the mites achieved an attachment force of 0.08 ± 0.12 mN with a safety factor of 51.47 ± 69.14. For this surface, statistically significant differences could also be found with all other tested surfaces in both force and safety factor (*p* < 0.001). The force values and calculated safety factors for 1 μm (total force: 0.007 ± 0.004 mN; safety factor: 4.34 ± 2.22) and 12 μm (total force: 0.011 ± 0.006 mN; safety factor: 7.19 ± 4.00) roughness, hydrophilic glass (total force: 0.011 ± 0.004 mN; safety factor: 7.23 ± 2.59) and hydrophobic plastic foil (total force: 0.009 ± 0.004 mN; safety factor: 7.23 ± 2.05) did not differ significantly (*p* > 0.001).

**Fig. 4:**
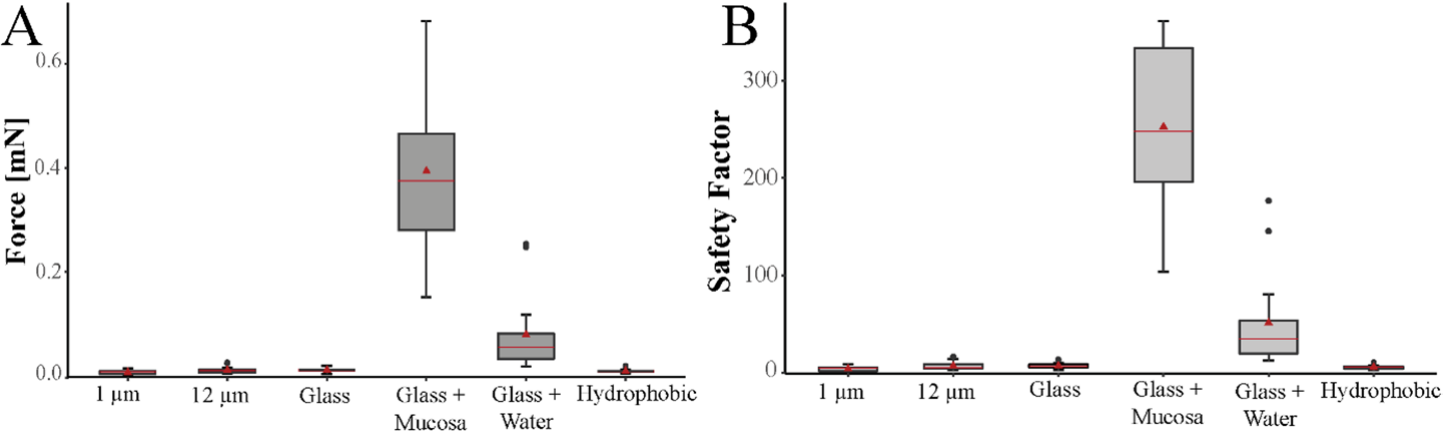
Attachment forces of hexapod larvae of *O. attenuata* on different surfaces. (**A**) Boxplots showing the total attachment force (in mN) on 1 μm and 12 μm roughness, on glass, on glass with fixed mucosa, on glass with water on its surface, and on hydrophobic plastic. (**B**) Boxplots showing the safety factor on the same surfaces as in A). In total, 15 individual mites (*n* = 15) were measured each three times on each surface and the mean of the three measurements was used. The boxes indicate 25th and 75th percentiles, the line within the boxes represents the median, and whiskers (error bars) define the 10th and 90th percentiles

## Discussion

*Orthohalarachne attenuata*, the respiratory mite of walruses and other pinnipeds, inhabits an exceptionally challenging and adverse environment: its host spends a substantial portion of its life foraging in the open sea, subjecting the mite to this demanding marine habitat with significant temperature fluctuations, salinity, pressure differences during dives, and hypoxia (Fay and Ray 1968; Fay and Burns 1988; Wiig et al. 1993; Leonardi et al. 2020). Continuous contact with its host is crucial for the parasitic mite’s survival, as it derives nutrition from the host’s nasal mucosa (Alonso-Farré et al. 2012; Reckendorf et al. 2019). Although Pinnipedia close their nostrils when diving to prevent water ingress (Dudzinski et al. 2009), which indicates that the mites are not exposed to direct currents during diving and can therefore only be classified as semi-aquatic, they must still anchor themselves securely in the nasal mucosa of their hosts to avoid unwanted expulsion from the respiratory tract, when the walruses engage in respiratory behaviors, such as sneezing or snorting (Furman and Smith 1973). To achieve this, the nasal mites securely anchor themselves in the nasal mucosa of their hosts (Helle and Sabelis 1985; Evans 1992; Fain 1994). However, they face the challenge of maintaining a secure attachment, while simultaneously navigating the slippery and moist nasal mucus, to facilitate nutrition, reproduction, and colonization of new hosts during walrus haul-outs (Furman and Smith 1973; Fay 1982).

In the course of our investigation, we observed that the larvae of *O. attenuata* exhibit two claws and a cushion-like pad (i.e., arolium) on each pretarsus, a configuration that represents the common basic arrangement of two claws and a median empodium found in most Mesostigmata (Evans 1992; Reckendorf et al. 2019; Shields et al. 2024). While this basic pretarsal design is widely conserved, the specific shape and functional properties of both claws and arolium in *O. attenuata* show distinct adaptations to its soft, mucus-covered substrate (Figs. 2 and 3). In general, tarsi of mites can consist of 1–3 claws, which are moved by two muscles (flexor and extensor). However, in Prostigmata, the central third claw is missing and replaced by discs or pads, whereas in Astigmata, there is only a single claw and a pad (Grandjean 1965). It has been shown that ecology and life style seem to play an important role in adaptations of claw morphology in mites, but the exact correlation between morphology and lifestyle remains unclear (Pfingstl 2023). In phoretic mites, such as *Paraleius leontonycha*, which attach to their hosts, the adult stage shows specifically curved claws for attaching to setae of the bark beetle for transport reasons (Knee 2017), while in intertidal mites, one single large claw with a very specific shape to withstand wave action and flooding has been reported (Pfingstl and Kerschbaumer 2022; Pfingstl 2023). In *O. attenuata* mites, which usually attach to the soft and slippery mucosa of walruses and otariids, the claws are comparably short and show the strongest curvature at their base (Fig. 3E). Thereby, they have a hook-like appearance, suggesting that these exoskeletal structures are used in a “hook and pull” manner (Pfingstl 2023), also shown experimentally by Heethoff and Koerner (Heethoff and Koerner 2007) in the oribatid mite *Archegozetes longisetosus*, which produces high pull-off and traction forces that increase with higher surface roughness due to claw interlocking. In contrast to this free-living mite, however, the nasal mite did not show significantly higher attachment forces on the rough artificial surfaces. The highest values were measured, when these larvae were clinging to the walrus mucosa (Fig. 4). This suggests that the claw shape in these mites seems to be particularly suitable for hooking into such soft, fibrous mucosa structure, whereas the claw of the oribatid mite *A. longisetosus* has a significantly stronger curvature towards the distal end of the claw and thus may be better suited for hooking into firmer, more rigid substrates such as soil (Heethoff and Koerner 2007; Pfingstl 2023).

Adhesive pads are present in various Arthropoda species including mites. In mites, they are for example known from several aboreal taxa and phoretic mites (Behan-Pelletier and Walter 2007; Pfingstl 2023; Sun et al. 2024). It is assumed that in these Acari taxa, such pads might contribute to better attachment on smooth surfaces, as seen for insects (Gorb and Gorb 2004), (Pfingstl 2023). Differences in pad morphology might be caused by different microhabitat preferences: *Adhaesozetes polyphyllos* (arboreal oribatid mite) for example shows a broad and dorsoventrally flattened pulvillus due to its association with smooth plant surfaces, while pulvilli can be completely lost during the development from larval to adult state in *Dendroeremaeus krantzi* (arboreal oribatid mite on forest trees) (Walter and Behan-Pelletier 1993; Behan-Pelletier et al. 2005). In the larva of *O. attenuata*, we observed arolia in all three leg pairs, forming a cushion-like structure appearing to be soft/flexible and possibly resilin-containing based on its blue autofluorescence (Fig. 2E–J). Their contact area increases visibly as soon as the arolium is pressed onto a smooth surface: an increase of the contact area presumably contributes to better adhesion to smooth surfaces (Gorb et al. 2001; Labonte et al. 2014).

In other Arthropoda, it has been described that their pretarsal adhesive organs are used together with their claws, as observed in grasshoppers (Slifer 1950), stick insects (Walther 1969), cockroaches (Arnold 1974; Frazier et al. 1999), and flies (Walker et al. 1985). In these insects, the adhesive pads are connected to the claws on the pretarsus by means of a tendon and the position of these pads is simply controlled by the claw position (Slifer 1950; Walther 1969; Arnold 1974; Walker et al. 1985; Frazier et al. 1999; Federle et al. 2001). However, it is noticeable that in *O. attenuata*, the arolium appears visually folded, when the claws are hooked into the nasal mucosa, and only appears extended, when the mite is on a smooth surface (Fig. 3; Porta et al. 2024). Therefore, this mechanism is reminiscent of that described for Hymenoptera (Snodgrass 1956), where it has been shown that the extension and inflation of the pad (arolium) is coupled with the retraction of the claw, as the unguitractor muscle pulls on the claws and the hairy planta is connected to the arolium (Federle et al. 2001, 2002). As a result, the arolium in the pretarsus of ants is activated through the contraction and relaxation of the claw flexor muscle (Federle et al. 2002). Usually, the claws first retract until reaching a position perpendicular to that of the tarsus, followed by an unfolding of the arolium, which also happens reversed in case of detachment. For this reason, the pad will not unfold at all if the claw gets caught on a rough surface beforehand and thus does not reach this perpendicular position (Snodgrass 1956). The pad therefore only unfolds on smooth surfaces. For unfolding, the tendon fixed to the unguitractor muscle is completely drawn back and hemolymph inflates the soft pad. Contrary to this, when the tendon is released, this results in a deflation of the pad, driven by the recoil of the elastic pad cuticle (Federle et al. 2001). We assume a similar mechanism in *O. attenuata*, as the pads seem to unfold in contact with smooth surfaces and the contact area was increased, when the tarsus was pressed onto the glass surface as demonstrated in the highspeed-video recordings (Fig. 3A–C). Furthermore, the contact angle with the surface is different depending on whether the mite attaches with its claws to the soft mucosa or with its pads to the smooth surface, supporting the idea of a folding mechanism based on the position of the claws. However, the pad of the mite differs morphologically from that of the Hymenoptera, which has a more lobe-like structure in bees and ants, so that the unfolding mechanism could probably still be somewhat different (Federle et al. 2001, 2002). In our SEM study, we could see that the arolium was folded very regularly and that the center of the pad in particular was pulled strongly inwards (Fig. 3D). This suggests that there may be another structure within this pad that “actively” pulls the pad inwards, which has not yet been described in Hymenoptera. However, as these are only assumptions so far, further investigations of the inner structures of the legs in these respiratory mites are necessary in the future to test this hypothesis.

When having a closer look at the pad surface, we were not able to detect any fluids that wet the surface as it has been observed in insect adhesive pads. Usually, adhesive secretions on insect smooth and hairy pads are very common and have been found in all insect groups studied to date, such as ants, stick insects, cockroaches, flies, beetles, and bugs (Edwards and Tarkanian 1970; Bauchhenß 1979; Ghazi-Bayat and Hasenfuss 1980; Ishii 1987; Federle et al. 2002). Also in other organisms, such as bats, tree frogs, spiders, and mites, adhesive fluids have been previously detected (Emerson and Diehl 1980; Riskin and Racey 2010; Dirks and Federle 2011). On the contrary, dry adhesion based on van der Waals forces between the substrate and hairy setae occurs in geckos and spiders (Autumn and Peattie 2002; Gasparetto et al. 2009). We assume that the adhesive pads of the respiratory mites do not secrete adhesive fluids as they are usually in contact with the wet mucus of their marine host animals, making additional wet secretion unnecessary. Instead, we suggest that the pad is always wettened by the mucus of the host and that the mite attachment might rely on Stefan adhesion, a viscosity-dependent hydrodynamic force, and capillary forces. Thereby, Stefan adhesion usually relies on viscous resistance in the fluid layer, while capillary adhesion is based on surface tension and curvature of the liquid meniscus at the interface between solid, fluid, and air (Emerson and Diehl 1980; Hanna and Barnes 1991; Nachtigall 2013). We therefore assume that in order to be able to walk on the wet surface, the mite changes the angle of the claw when detaching the pretarsus so that the pad is retracted as described above and that air can get between the liquid film and the pad due to the lack of tension and the probable resulting formation of folds on the surface of the pad: in this way, detachment is facilitated. Our assumption regarding wet adhesion in the mites could also explain the comparatively high attachment forces in the experiments on wet glass: the claws could not be used on the smooth surface and the water film could have improved adhesion to the pads based on the capillary forces (assuming that the pad was not completely under water, but only a thin film of water was on the glass surface) and Stefan adhesion (Fig. 4). However, we cannot rule out the possibility that the water film was not also drawn over the entire body of the mite during the rotation of the centrifuge, so that this could also have had an influence on the adhesion.

However, in our centrifugal force tests, by far the highest values for attachment were found, when the mite was able to either anchor itself with its claws in its natural substrate, namely the walrus mucosa (safety factor: 468.3 in individual measurements), or adhere to the wet glass surface with its pads (safety factor: 208.47 in individual measurements). Comparatively high values were achieved, for example, by the ant *Atta cephalotes* with a safety factor of about 350 (Pattrick et al. 2018), while most other insects, with the exception of parasitic species (Petersen et al. 2018; Büscher et al. 2022; Preuss et al. 2024), showed significantly lower values (Bußhardt et al. 2014; Burack et al. 2022; Salerno et al. 2022). For mites, force measurement have been implemented in the oribatid mite *A. longisetosus*, revealing high pull-off forces with safety factors of 1800 and strong traction forces with safety factors of 530 (Heethoff and Koerner 2007), which by far exceed the forces we have obtained in our respiratory mites. However, in our experiments we only measured hexapod larvae with maximum six legs in contact, whereas in the other experiments, adults with eight legs have been used. It would therefore be highly interesting to measure less mobile octopod stages, i.e. nymph and adult stages of *O. attenuata* for comparison, as eight legs would presumably achieve a higher attachment force.

It would be also interesting to observe, whether the gait of adult *O. attenuata* mites changes as they no longer have six legs but rather eight. However, it is well possible that the tripod gait is particularly well suited to the agile lifestyle of these larvae, as these parasitic stages are known to be very mobile and tend to reside more rostrally, in the upper part of their host’s respiratory tract. This positioning likely facilitates transmission between hosts, when the opportunity arises (Furman and Smith 1973; Geraci and St. Aubin 1987). In contrast, octopod adult *O. attenuata* are more sessile and are typically found deeper within the respiratory tract, predominantly infesting the nasopharyngeal mucosa (Geraci and St. Aubin 1987). Accordingly, agility is presumably much less important for these stages than secure and continuous attachment to the host tissue. The presence of eight legs in adults should therefore not be interpreted as an adaptation to agility, but rather as the retention of the ancestral chelicerate body plan, which may nonetheless provide improved stability and attachment within the host’s respiratory passages.

These observations are consistent with the morphological findings reported by Porta et al. (2024), who provided a detailed comparative description of the ambulacral structures in both larval and adult *O. attenuata*. Their study revealed marked differences between developmental stages, including differences in claw curvature (more strongly curved and grooved in larvae, straighter and longer in adults), in the development of the paradactyli (flipper-shaped and partially covering the pretarsus in larvae, reduced in adults), and in the relative size of the pad itself, which is more prominent and retractable in larvae but more compact in adults. Porta et al. (2024) interpreted these modifications as adaptations to contrasting life modes: enhanced mobility and host transfer in larvae versus permanent attachment and reduced locomotion in adults. Our functional and biomechanical analyses support and extend this interpretation by demonstrating how the structural configuration of the claws and the resilin-rich arolium enables efficient attachment to the soft, mucus-covered surfaces of the walrus nasal cavity. Taken together, both studies highlight how the conserved mesostigmatid ambulacral design can be fine-tuned to meet the demands of highly specialized host-associated environments.

To conclude the above results, we have observed that *O. attenuata* mites show different ways of attachment depending on the substrate they are exposed to. On their native substrate, they exclusively rely on their claws for clinging to the fibrous material of the mucosa. On smooth surfaces, both dry and wet, they only use their adhesive pads and in the case of wet smooth substrates, probably rely on Stefan adhesion and/or capillary forces. On microrough surfaces (e.g., 1 μm and 12 μm roughness), the animals likely engage their claws and/or arolia to contact the substrate, but less effectively than on smoother or more textured surfaces, as suggested by our force measurements. Presumably, a complex mechanism based on different muscles, tendons, the angle of attack of the legs, flexible cuticle in the pads and hemolymph pressure can regulate which of their attachment devices forms contact with corresponding surface. This mechanism should be investigated in more details in the future including octopod stages. Generally, respiratory mites’ attachment devices can be used almost universally, due to their adaptation to the challenging habitat in order to successfully survive as parasites on their host surfaces surrounded by the marine environment, suggesting a successful coevolution with their mammalian marine hosts. Thus, halarachnid mites may offer inspirations for technical applications for secure and rapidly adaptable attachment on wet surfaces.

## Supplementary Information

Below is the link to the electronic supplementary material.

Supplementary Material 1

Supplementary Material 2

Supplementary Material 3

Supplementary Material 4

Supplementary Material 5

Supplementary Material 6

Supplementary Material 7

Supplementary Material 8

Supplementary Material 9

## Data Availability

The data that support the findings of this study are available in the Supplementary Material.
