# Supplementary material for: Locomotion and attachment mechanisms of the respiratory mite Orthohalarachne attenuata
Source: Exp Appl Acarol. 2025 Dec 2;95(4):66. doi: 10.1007/s10493-025-01094-8 (PMC12672789; doi:10.1007/s10493-025-01094-8)
Supplement: Supplementary file 1 — Supplementary Material 1 [file 10493_2025_1094_MOESM1_ESM.docx]

**Suppplementary Material**

**S1)** High-speed video recording of an *O. attenuata* larva on glass filmed with a reflection interference contrast microscope (RICM) from ventral side at 5400 fps.

**S2)** Larva of *O. attenuata* moving freely in a Petri dish at original speed filmed from dorsal side.

**S3)** Contact angle measurement data for water on hydrophobic plastic foil.

**S4)** Live-image taken during contact angle measurement of water on hydrophobic plastic foil.

**S5)** Contact angle measurement data for water on hydrophilic glass.

**S6)** Live-image taken during contact angle measurement of water on hydrophilic glass.

**S7)** Force measurement data from centrifugal force measurements for larvae on different surfaces (1 µm (roughness) polishing paper; 12 µm (roughness) polishing paper, (iii) hydrophilic glass, (iv) hydrophobic plastics; hydrophilic glass with water on its surface; polished glass with a piece of walrus mucus).

**S8)** R-scripts used for statistics and graphs.
