# Supplementary figures and images for: Locomotion and attachment mechanisms of the respiratory mite Orthohalarachne attenuata
Source: Exp Appl Acarol. 2025 Dec 2;95(4):66. doi: 10.1007/s10493-025-01094-8 (PMC12672789; doi:10.1007/s10493-025-01094-8)

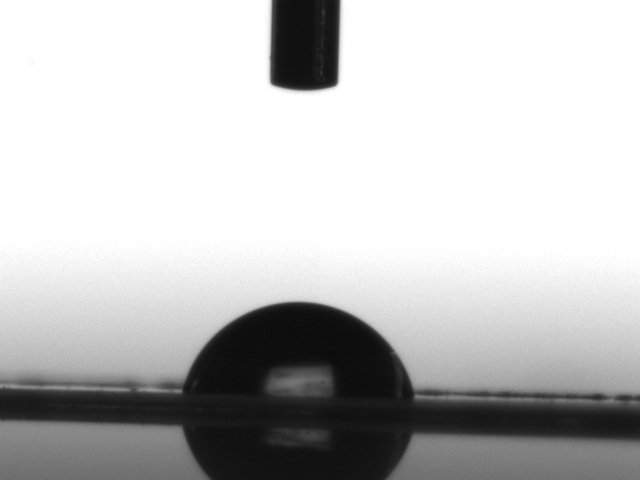

Supplement: Supplementary file 5 — Supplementary Material 5 [file 10493_2025_1094_MOESM5_ESM.bmp]

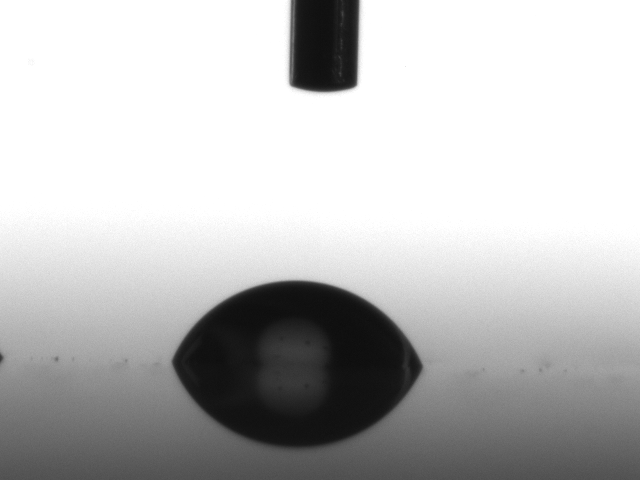

Supplement: Supplementary file 7 — Supplementary Material 7 [file 10493_2025_1094_MOESM7_ESM.bmp]
