# Supplementary material for: Locomotion and attachment mechanisms of the respiratory mite Orthohalarachne attenuata
Source: Exp Appl Acarol. 2025 Dec 2;95(4):66. doi: 10.1007/s10493-025-01094-8 (PMC12672789; doi:10.1007/s10493-025-01094-8)
Supplement: Supplementary file 9 — Supplementary Material 9 [file 10493_2025_1094_MOESM9_ESM.docx]

############################################################################################################################################

This R-script is part of the manuscript "Locomotion and Attachment Mechanisms of the Walrus Respiratory Mite *Orthohalarachne attenuata*"

+++++++++ Boxplot Force in mN +++++++++++++

###########################################################################################################################################

# Clear workspace

rm(list = ls())

library(ggplot2)

# Import dataset

library(readxl)

S7_force_measurements <- read_excel("D:/Manuskripte/Mite/S7_force_measurements.xlsx",

sheet = "Summary")

View(S7_force_measurements)

attach(S7_force_measurements)

# Make boxplot

pd = position_dodge(width = 1.1)

p <- ggplot(S7_force_measurements, aes(x=Treatment, y=F_total_in_mN)) +

stat_boxplot(geom='errorbar', position = pd, width=0.1) +

geom_boxplot(width = 0.3, position=position_dodge(width = 1.1)) +

stat_summary(fun = mean, geom = "point", color = "firebrick", shape = 17, size = 2, position = position_dodge(width = 1.1)) +

scale_fill_manual(values=c("#999999", "#E69F00")) +

theme_classic() +

labs(y = "Force in mN") +

theme(plot.title = element_text(hjust = 0.5, size = 16, face = "bold"), plot.caption = element_text(hjust = 0, size = 10), plot.tag = element_text(size = 16, face = "bold")) # adjust title and caption position

p

############################################################################################################################################

This R-script is part of the manuscript "Locomotion and Attachment Mechanisms of the Walrus Respiratory Mite *Orthohalarachne attenuata*"

+++++++++ Boxplot Safety Factor +++++++++++++

###########################################################################################################################################

# Clear workspace

rm(list = ls())

library(ggplot2)

# Import dataset

library(readxl)

S7_force_measurements <- read_excel("D:/Manuskripte/Mite/S7_force_measurements.xlsx",

sheet = "Summary")

View(S7_force_measurements)

attach(S7_force_measurements)

# Make boxplot

pd = position_dodge(width = 1.1)

p <- ggplot(S7_force_measurements, aes(x=Treatment, y=mean_sf)) +

stat_boxplot(geom='errorbar', position = pd, width=0.1) +

geom_boxplot(width = 0.3, position=position_dodge(width = 1.1)) +

stat_summary(fun = mean, geom = "point", color = "firebrick", shape = 17, size = 2, position = position_dodge(width = 1.1)) +

scale_fill_manual(values=c("#999999", "#E69F00")) +

theme_classic() +

labs(y = "Force in mN") +

theme(plot.title = element_text(hjust = 0.5, size = 16, face = "bold"), plot.caption = element_text(hjust = 0, size = 10), plot.tag = element_text(size = 16, face = "bold")) # adjust title and caption position

p

########################################################################################################################################

This R-script is part of the manuscript "Locomotion and Attachment Mechanisms of the Walrus Respiratory Mite *Orthohalarachne attenuata*"

+++++++++ Krsukal-Wallis Test Safety Factor +++++++++++++

########################################################################################################################################

# Clear workspace

rm(list = ls())

library(ggplot2)

library(tidyverse)

library(ggpubr)

library(rstatix)

# Import dataset

library(readxl)

S7_force_measurements <- read_excel("D:/Manuskripte/Mite/S7_force_measurements.xlsx", sheet = "Statistics")

View(S7_force_measurements)

attach(S7_force_measurements)

# Data preparation

S7_force_measurements <- S7_force_measurements %>%

gather(key = "Treatment", value = "sf", 1µm, 12µm, Glass, Hydrophob, Glass_Mucos, Glass_Water) %>%

convert_as_factor(Animal)

data.frame(S7_force_measurements, 6)

# Summary statistics

S7_force_measurements %>%

group_by(Treatment) %>%

get_summary_stats(sf, type = "mean_sd")

# Check for outliers

S7_force_measurements %>%

group_by(Treatment) %>%

identify_outliers(sf)

# Normality assumption

S7_force_measurements %>%

group_by(Treatment) %>%

shapiro_test(sf) # not normally distributed -> Kruskal Wallis

# Kruskal-Wallis ANOVA on ranks

res.kruskal <- S7_force_measurements%>% kruskal_test(sf ~ Treatment)

res.kruskal

# Due to significant difference: Post-hoc-test with Dunn's (Multiple pairwise comparisons)

pwc2 <- S7_force_measurements %>%

dunn_test(sf ~ Treatment, p.adjust.method = "bonferroni")

pwc2

print(as_tibble(pwc2), n=50)

#########################################################################################################################################

This R-script is part of the manuscript "Locomotion and Attachment Mechanisms of the Walrus Respiratory Mite Orthohalarachne attenuata"

+++++++++ Krsukal-Wallis Total Force in mN +++++++++++++

#########################################################################################################################################

# Clear workspace

rm(list = ls())

library(ggplot2)

library(tidyverse)

library(ggpubr)

library(rstatix)

# Import dataset

library(readxl)

S7_force_measurements <- read_excel("D:/Manuskripte/Mite/S7_force_measurements.xlsx", sheet = "Statistics")

View(S7_force_measurements)

attach(S7_force_measurements)

# Normality assumption -> not normally distributed -> < 0.05

shapiro.test(S7_force_measurements$mean_sf)

# Data preparation

S7_force_measurements <- S7_force_measurements %>%

gather(key = "Treatment", value = "F", 1µm, 12µm, Glass, Hydrophob, Glass_Mucos, Glass_Water) %>%

convert_as_factor(Animal)

data.frame(S7_force_measurements, 6)

# Summary statistics

S7_force_measurements %>%

group_by(Treatment) %>%

get_summary_stats(F, type = "mean_sd")

# Check for outliers

S7_force_measurements %>%

group_by(Treatment) %>%

identify_outliers(F)

# Normality assumption

S7_force_measurements %>%

group_by(Treatment) %>%

shapiro_test(F) # not normally distributed -> Kruskal Wallis

# Kruskal-Wallis ANOVA on ranks

res.kruskal <- S7_force_measurements%>% kruskal_test(F ~ Treatment)

res.kruskal

# Due to significant difference: Post-hoc-test with Dunn's (Multiple pairwise comparisons)

pwc2 <- S7_force_measurements %>%

dunn_test(F ~ Treatment, p.adjust.method = "bonferroni")

pwc2

print(as_tibble(pwc2), n=50)
